# Supplementary figures and images for: Genome-wide analysis of the lignin toolbox for morus and the roles of lignin related genes in response to zinc stress
Source: PeerJ. 2021 Aug 6;9:e11964. doi: 10.7717/peerj.11964 (PMC8351576; doi:10.7717/peerj.11964)

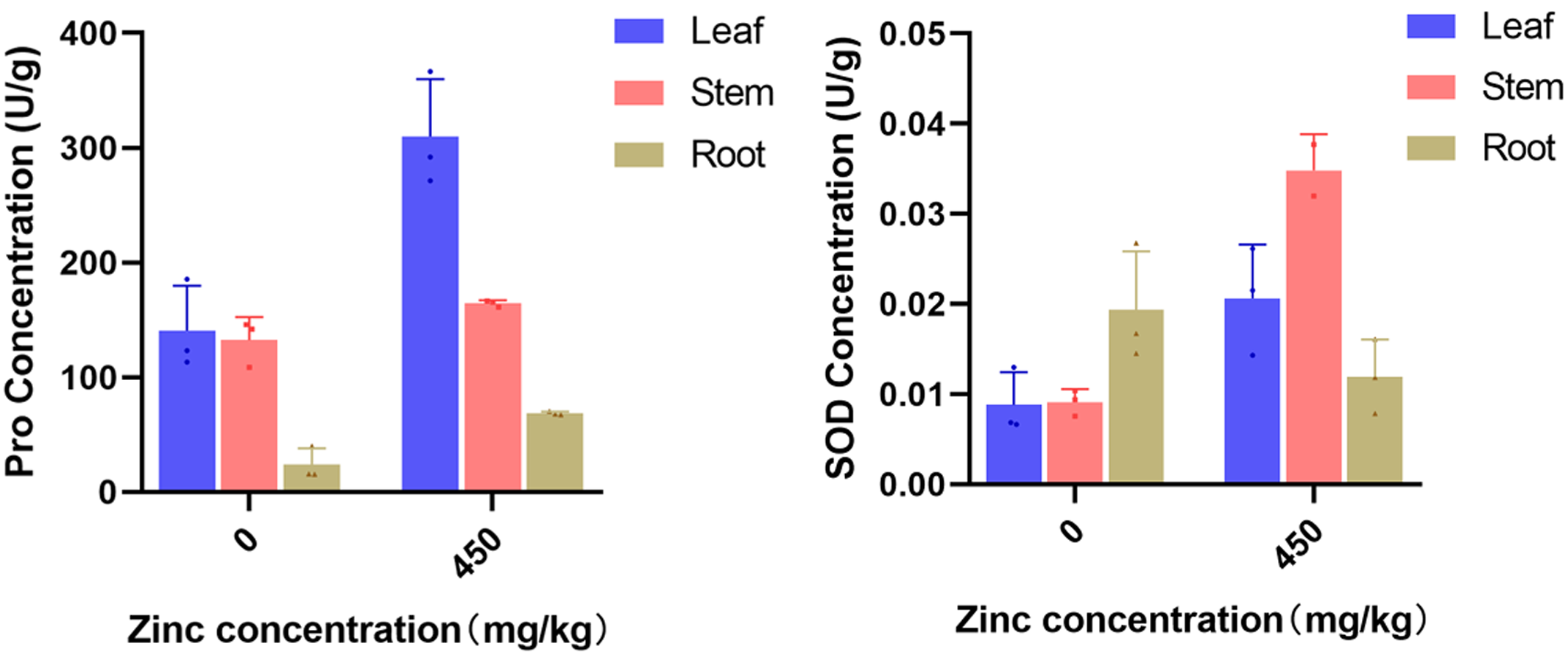

Supplement: Supplemental Information 1 [file peerj-09-11964-s001.png]

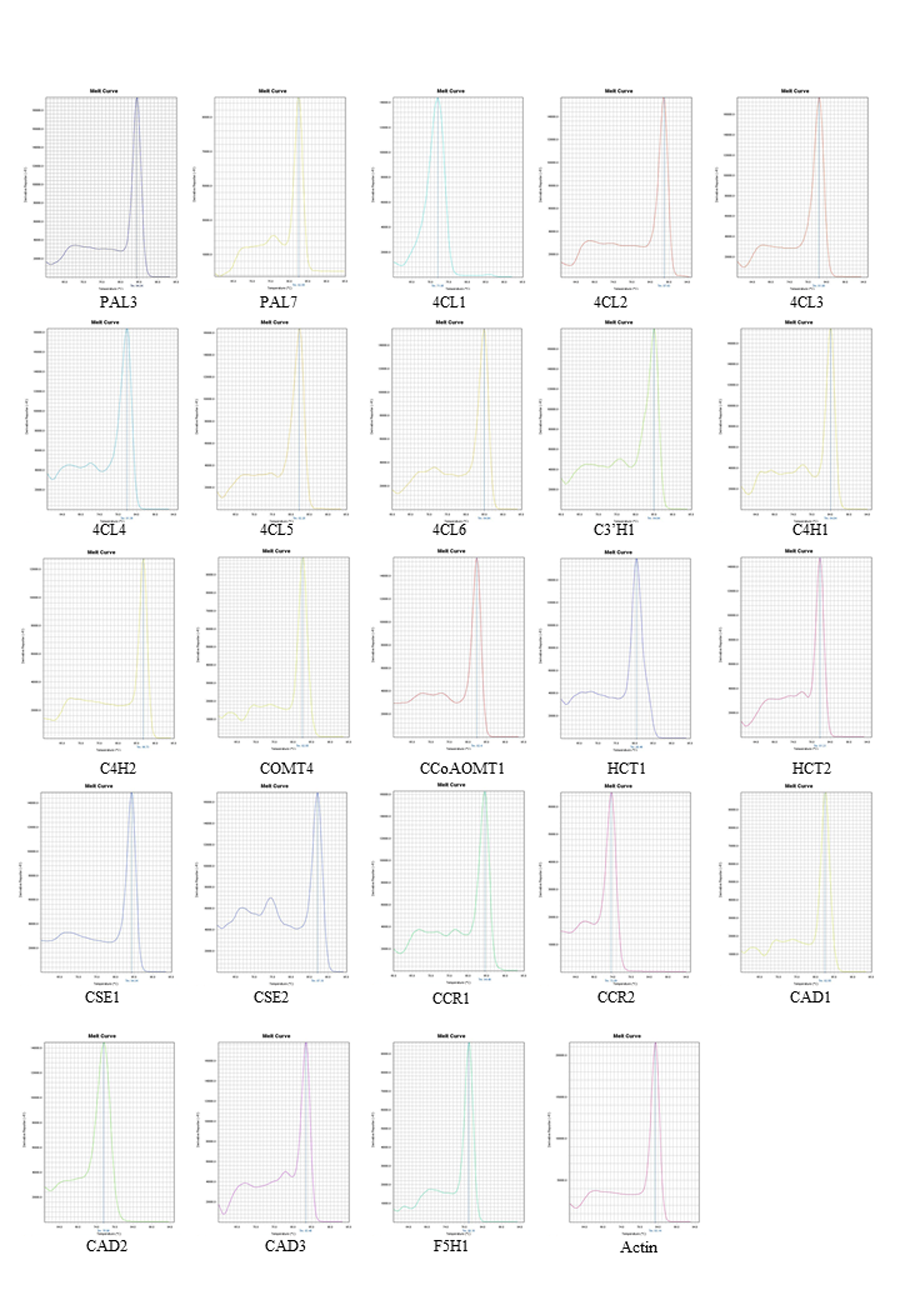

Supplement: Supplemental Information 2 [file peerj-09-11964-s002.png]

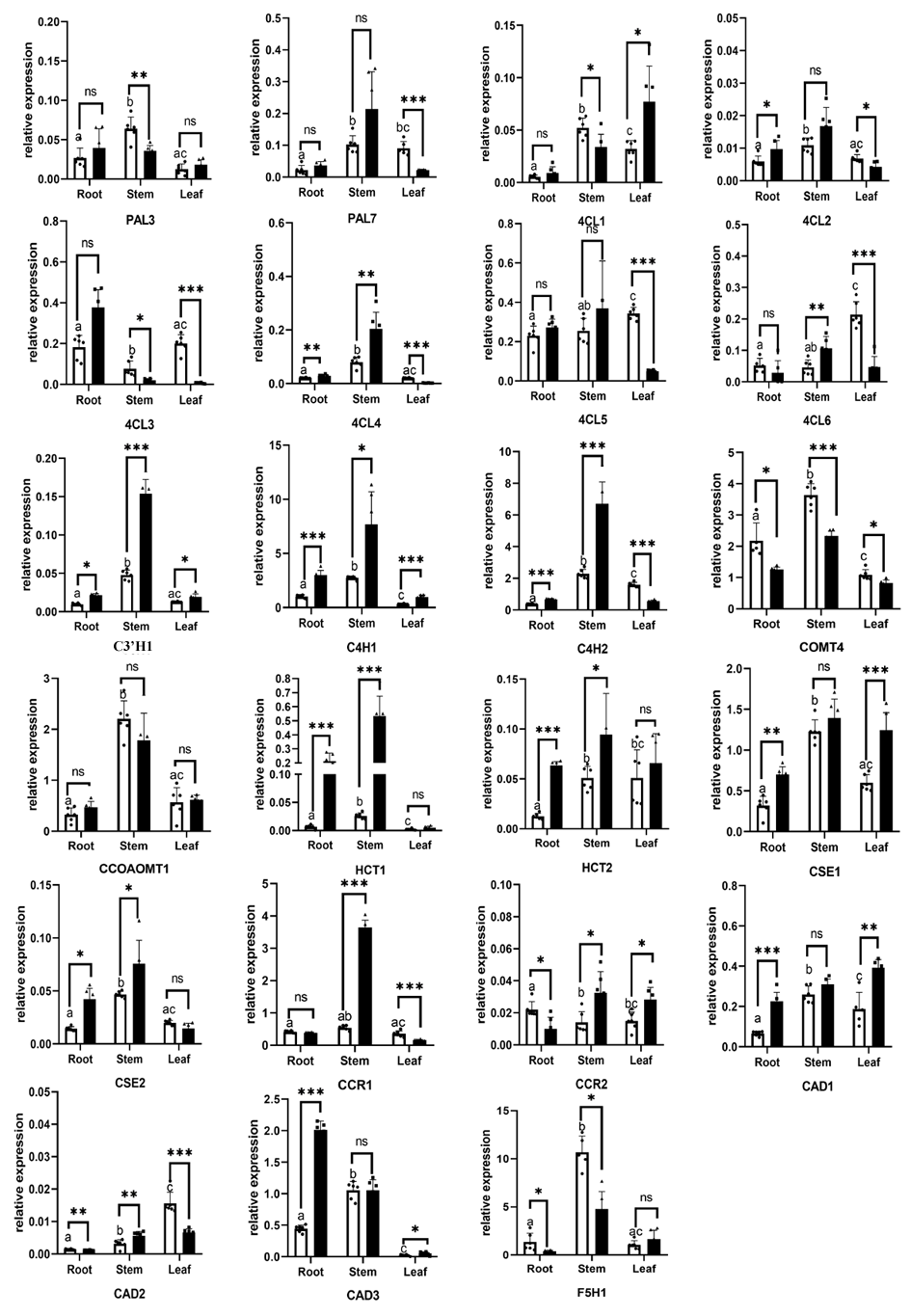

Supplement: Supplemental Information 3 [file peerj-09-11964-s003.png]

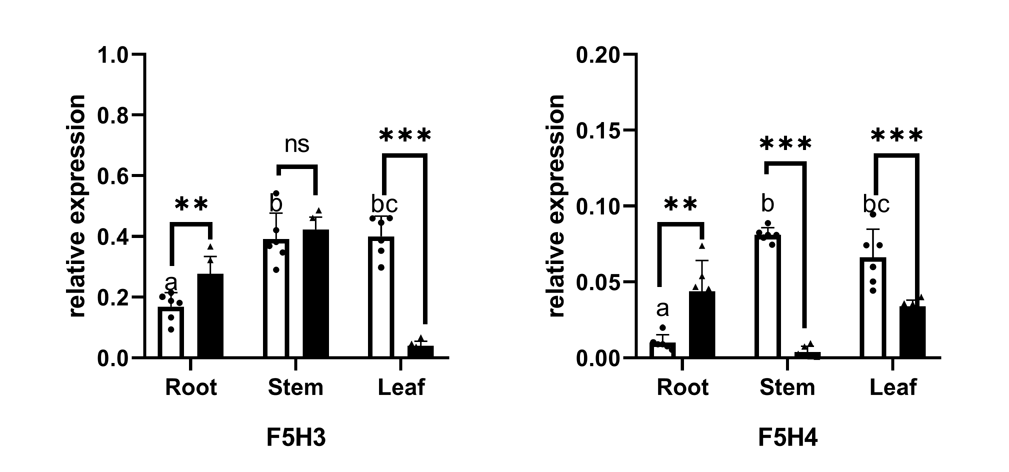

Supplement: Supplemental Information 4 [file peerj-09-11964-s004.png]

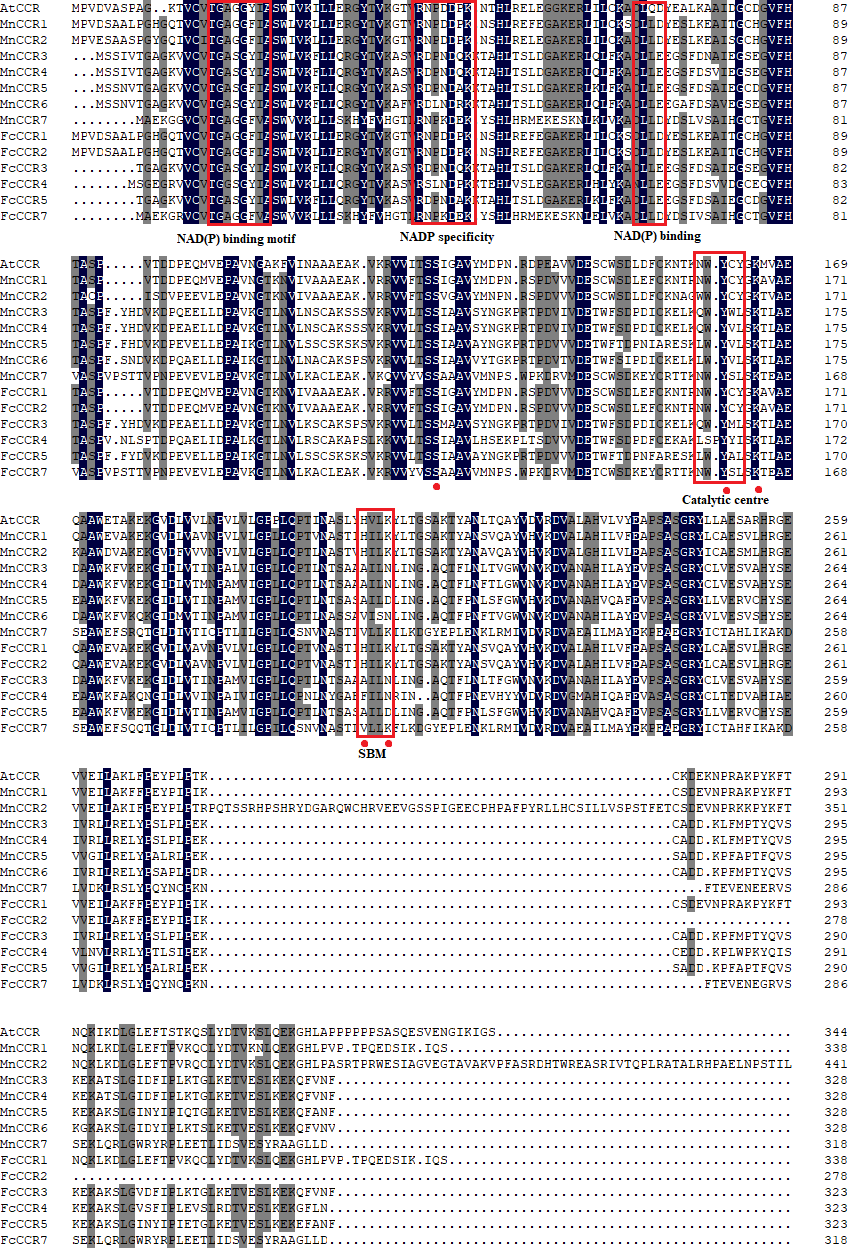

Supplement: Supplemental Information 5 — The motifs were marked in red box. [file peerj-09-11964-s005.png]

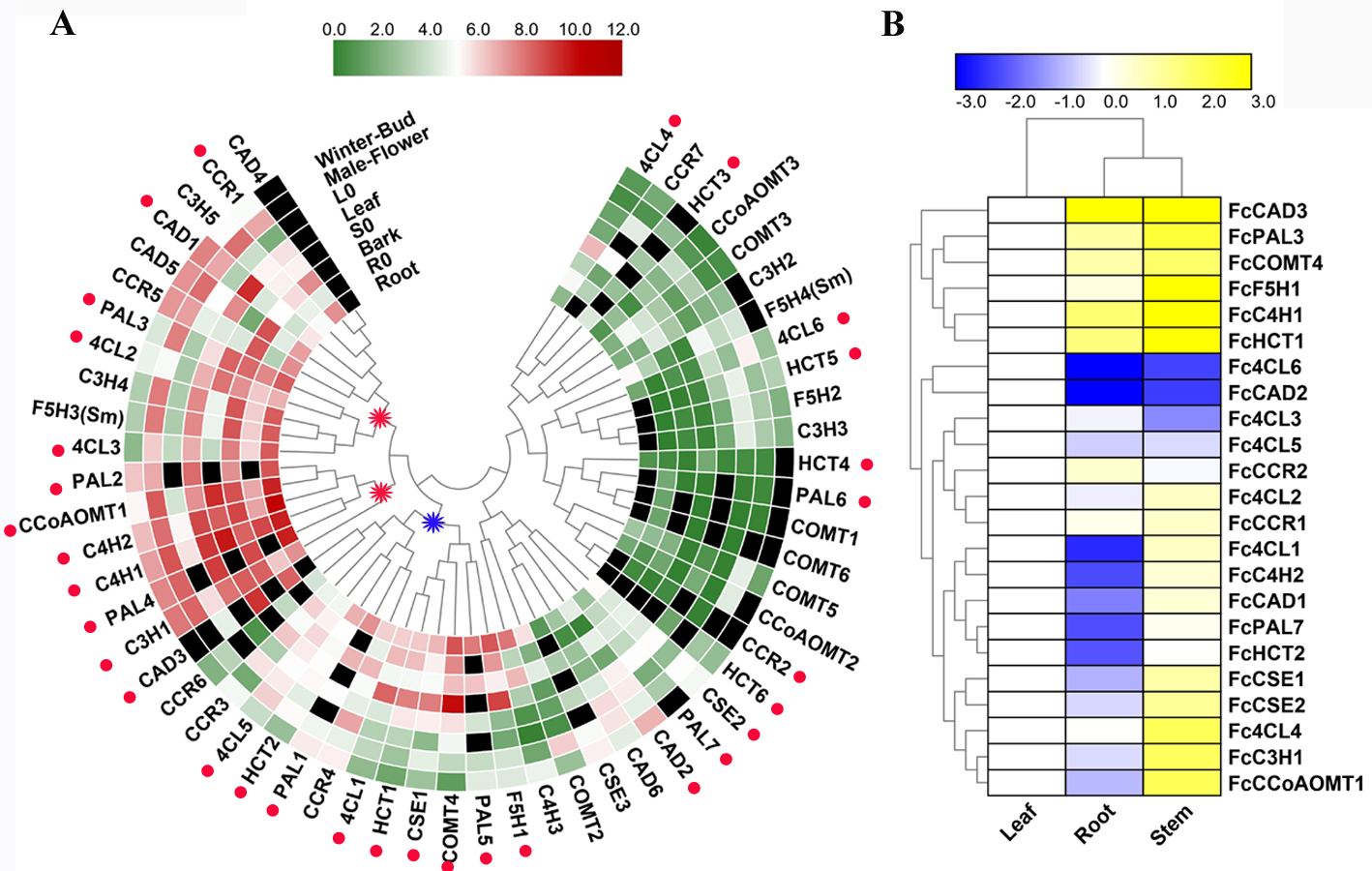

Supplement: Supplemental Information 6 — (A) Hierarchical clustering of expression profiles of 56 candidate genes based on transcriptome data in mulberry; Blue star indicated the cluster I and red star indicate the cluster II. Red full circles indicated the bona fide clade genes (B). Expression profiles of 23 bona fide clade genes in Fengchi. [file peerj-09-11964-s006.png]
